# Catherine L. Bagwell and Michelle E. Schmidt: Friendships in childhood and adolescence: New York: The Guilford Press, 2011 ISBN 978-1-60918-646-3

**DOI:** 10.1007/s00787-012-0268-7

**Published:** 2012-03-21

**Authors:** Frits Boer

**Affiliations:** Academic Medical Centre, Amsterdam, The Netherlands

There is no word for *friend* in the language of the Yucatec Maya, but this is so exceptional that it only underscores the universal nature of the construct of friendship. A construct that is easily understood intuitively, but difficult to define, because of its multifaceted nature. Catherine Bagwell and Michelle Schmidt have taken it upon them to unravel the construct of friendship, to establish its developmental significance, and to find out how the context of friendship is significant for adjustment and well-being. This has led to a comprehensive and excellent volume.

Friendship in Childhood and Adolescence consists of nine chapters, divided into four parts. Part I, The Nature of Friendship, defines friendship and shows how friendship can be studied. The authors do not only discuss the developmental psychological literature on friendship but also deal with the contributions of sociology, anthropology, attachment theory, ecological systems theory and learning theory. Two important conclusions of this part are that we know much about the nature of children’s friendships, but little about the processes that occur within children’s interactions. Furthermore there has been a long-standing assumption that friendships contribute to positive adjustment. Only recently more attention is paid to the potential negative aspects of friendship, with conflict as the only dimension. The authors call for attention to features such as rivalry, inequality, deviancy training, jealousy and social aggression between friends.

Part II, The normative experience of friendship, discusses the developmental significance of friendship in childhood and adolescence. The four characteristics that appear most important for the role friendship plays in development are that friendships are (1) voluntary, (2) reciprocal, (3) based on a strong affective tie, and (4) exist between children who are at similar developmental levels. These characteristics are used to determine the developmental significance in the social, emotional and cognitive domain and with regard to psychosocial adjustment. Obviously childhood and adolescence bring different developmental tasks with them, and one of the tasks of adolescence is to balance the relationships with parents and peers. Popular media often portray adolescence as a period in which peers and friends completely overshadow the influence of parents. Research shows a more complex picture in which parent and peer relationships (including friendships) both serve important functions, some overlapping and some distinct. For instance, it may be that parents provide security, and best friends and romantic partners meet other attachment needs such as comfort, support and reassurance.

After reviewing this literature, the authors express the need to be able to draw sharper, more nuanced conclusions rather than sweeping statements about its importance. This means expanding our understanding of moderator variables, without assuming that friendship has the same significance in all situations and for all children.

Part III, Individual differences in the experience of friendship, discusses the individuals within a friendship and friendship quality. This part also deals with ‘the dark side’ of friendship, the contribution of friends to one another’s antisocial behaviour. It refers to an interesting study by one of the authors (CB) about ‘temptation talk’, that is the exploration in discussions between friends of potential rule violations, such as cheating on a word scramble task using the answer key. This kind of talk helps children in middle childhood to mutually negotiate and figure out the norms of the friendship. It turns out that aggressive and nonaggressive friends do not differ in the amount of time they spent in this kind of talk. What distinguishes the two groups is that nonaggressive friends tend to engage in temptation talk and then return to normative topics. For the aggressive friends, in contrast, this exploration is more likely to lead them to actual rule-breaking behaviour.

It does not surprise to read that not having friends is a risk factor for depression in adolescence, but the opposite is true as well: having friends who are depressed themselves may be just as significant a risk factor for depression. A process that may contribute to this last association is *corumination*: rehashing problems and dwelling on negative affect with a friend.

The final three chapters in Part IV are (1) on cultural context, and cross-cultural similarities and differences, (2) interventions to help children who have trouble in forming and maintaining positive friendships, and (3) a concluding chapter with a conceptual framework for friendship and its significance for children and adolescents. The conceptual framework is a successful attempt to integrate the wide range of issues discussed in the book, and will be very helpful in generating questions for further research.

Catherine Bagwell and Michelle Schmidt are not the editors but the co-authors of this book. They have succeeded in synthesising their writing so well that their book reads like a one author volume. This even applies to Chap. 9 about friendship and culture, where Emily Jenchura serves as the third author. Where edited books often have chapters of a very different level, this book shows constant quality. It is clearly written and provides an up-to-date and critical account of the research literature in a language that makes it suitable for use as a textbook as well. The reader is well-served with regular summaries within chapters and concluding paragraphs after each chapter, in which future research directions are pointed out as well.

There are two topics that did not receive the attention I had hoped they would. As a child psychiatrist I was struck by the fact that no special attention is paid to children and adolescents with autism spectre disorders (ASD). Although the book does not explicitly treat psychopathology, it does pay attention to children with anxiety disorders and with conduct disorders. There is also a discussion of social cognitive functioning, including the development of theory of mind. It would be of interest to see how the social cognitive style of children with ASD intersects with the development of friendship. Something to consider for a next edition. The second topic will certainly be dealt with extensively in a future edition. This is the role of social media, such as e-mail, text messages, and online social network applications (e.g., Facebook). Bagwell and Schmidt acknowledge the importance of electronic communication in the friendships of children and adolescents, but as they write, research has not yet caught up with these trends. Undoubtedly this will change within the next 5 years or so. At this very moment, the landscape of peer relations and friendships is under total reconstruction by the internet. I look forward to a new edition of this book in a couple of years, to find out what the new landscape looks like.

